# Tremorgenic mycotoxin poisoning in a dog: A case report

**DOI:** 10.17221/82/2023-VETMED

**Published:** 2023-12-29

**Authors:** Tereza Novotna, Barbora Sitarova, Zlata Hoskova, Vera Vaibarova, Zbynek Dzuman, Jana Hajslova, Vojtech Skupien, Zdenka Svobodova

**Affiliations:** ^1^Department of Animal Protection and Welfare and Veterinary Public Health, Faculty of Veterinary Hygiene and Ecology, University of Veterinary Sciences Brno, Brno, Czech Republic; ^2^DRAVET, Veterinary Clinic, Drásov, Czech Republic; ^3^Veterinary Clinic with Emergency Service MVDr. Lubomír Hošek, Brno, Czech Republic; ^4^Department of Infectious Diseases and Microbiology, Faculty of Veterinary Medicine, University of Veterinary Sciences Brno, Brno, Czech Republic; ^5^Department of Food Analysis and Nutrition, University of Chemistry and Technology, Prague, Czech Republic; ^6^Faculty of Veterinary Medicine, University of Veterinary Sciences Brno, Brno, Czech Republic

**Keywords:** chromatography, paxilline, *Penicillium* sp., penitrem A, roquefortine C

## Abstract

An eleven-year-old Pit Bull Terrier was presented to the veterinary practice with an acute onset of whole-body seizures. The clinical signs developed in a garden where the dog was kept that morning. There was a suspicion of tremorgenic mycotoxin poisoning by compost as the dog had vomited parts of compost right before the onset of the seizures and there was a pile of compost located in the garden. The dog underwent immediate decontamination following supportive treatment and recovered fully within 24 h of intensive care. The samples of the vomit and parts of the compost were cultivated. In the sample of the vomit, *Penicillium* sp*.* was found. Subsequently, tremorgenic mycotoxins paxilline, penitrem A and roquefortine C were determined chromatographically at significant concentrations in the vomit and a growth medium with cultivated *Penicillium* sp*.* The aim of this work is to describe the complex therapeutic and diagnostic approach to the patient with a suspected tremorgenic mycotoxin poisoning where a combination of mycological and chromatographic analyses was used to confirm the diagnosis. To the best of our knowledge, this is the first confirmed case of canine tremorgenic mycotoxicosis in the Czech Republic and the first reported case of paxilline poisoning in a dog.

Tremorgenic mycotoxins are secondary metabolites produced by fungi from genera *Penicillium, Aspergillus*, *Claviceps* and *Neotyphodium.* Specifically, mycotoxins with tremorgenic ability include penitrems, roquefortine A, lolitrems, paspalinine, paxilline, verruculogen and other substances. After ingestion, they are quickly absorbed in the gastrointestinal tract. In addition, tremorgenic mycotoxins containing indol (penitrem A, roquefortine A, lolitrems, paspalinine, paxilline, verruculogen) are able to pass through the blood-brain barrier and affect synapses in the central nervous system (Selala et al. 1989; Evans and Gupta 2018). Therefore, these mycotoxins are classified as neurotoxins (Puschner 2009; Svobodova et al. 2017). The exact mechanism of their action is unknown, but interference with the spontaneous release of transmitter amino acids in central and peripheral synapses has been described (Young et al. 2003). The majority of intoxications in dogs or cats are caused by ingestion of mouldy food containing penitrem A and roquefortine C, mycotoxins most commonly associated with *Penicillium crustosum*, which is responsible for food spoilage. Penitrem A has been the most studied of the tremorgenic mycotoxins. In the CNS it increases the release of aspartate and glutamate, but also increases levels of GABA and decreases levels of glycine. As aspartate and glutamate are the main excitatory neurotransmitters, their increase leads to convulsions. Penitrem A also affects Purkinje cells. Exposure to this mycotoxin causes their degeneration and necrosis in cerebellar granular cell layers. Roquefortine C is reported to induce paralysis in several species, although its tremorgenic potential is low. Tremorgenic mycotoxins are frequently found in mould-ripened cheese, meat, rice, cereals, eggs, nuts, fruits, processed foods, dog food, refrigerated food, refuse, or compost (Sobotka et al. 1978; Arp and Richard 1981; Evans and Gupta 2018). The focal case of this article will describe a poisoning most likely caused by the ingestion of material contained in compost.

## Case description

### HISTORY AND CLINICAL EXAMINATION

An eleven-year-old 24 kg intact female American Pit Bull Terrier was presented to the local veterinary practice with an acute onset of generalised clonic seizures and facial myoclonus. The dog was fully conscious, tachypnoeic, and tachycardic with hyperaemic mucous membranes and a capillary refill time of 1.5 seconds. Rectal temperature was 38.9 °C. In-house blood work revealed mild hyperglycaemia, hypophosphataemia and thrombocytosis, other haematological and biochemical values were within the reference ranges of the veterinary analyser for the particular animal species and its age.

The owner found the dog less than one hour before. The dog was lying and vomiting in the garden where a compost was situated. Shortly after that, the seizures developed. In its vomit, pieces of overripe bananas from the compost were found. The owner took the dog to the veterinary practice with a sample of the vomit right after the seizures appeared. The dog had not shown any neurological issues before, it was regularly vaccinated and dewormed. It had been monitored for multiple nodules on the mammary gland. According to the history and sudden onset of the neurological symptoms, tremorgenic mycotoxin poisoning was suspected. The sample of the vomit was stored refrigerated for laboratory analysis at –20 °C.

### THERAPY AND OUTCOME

The dog was initially treated with activated charcoal (1 g/kg p.o.), diazepam (0.4 mg/kg i.v.), and fluids (Ringer lactate, 6 ml/kg/hr i.v.). After the administration of diazepam, the seizures subsided, but mild fasciculations in the face were still apparent. Due to the persistent neurological signs, the dog was referred to the veterinary hospital for intensive monitoring and treatment. At the hospital admission, the dog was alert but weak and ataxic. There was miosis on both pupils and the palpebral and pupillary light reflexes were slowed. Blood tests were repeated on hospital admission using an in-house veterinary analyser and revealed only mild neutrophilia and lymphocytopaenia.

The dog was continued on fluid therapy (Ring-erfundin + glucose, 4 ml/kg/hr i.v.) and humic acids were administered (167 mg/kg p.o.). The dog vomited again during the night and then maropitant (1.2 mg/kg i.v.) and famotidine (1.2 mg/kg i.v.) were administered. The next morning the clinical status improved rapidly, there occurred neither further fasciculations nor vomiting. On neurological examination the gait was coordinated. Due to the fast recovery, the dog was discharged in the afternoon with humic acids (167 mg/kg p.o. b.i.d.) and omeprazole (0.83 mg/kg p.o. s.i.d.) medication. The owners were advised to prevent it from accessing the compost. One week after the incident the dog was bright, alert, and responsive without any ongoing neurological signs. By the time an investigation of the garden where the dog had been kept was performed. In the garden, there was a compost pit where a few overripe bananas were found. The compost was not isolated so the dog was able to access it. The owner claimed the dog used to spend the whole day in the garden and due to its good appetite and a tendency to eat grass, it is possible that it ate a part of the compost. Except for the material from the compost, no other possible sources of the poisoning were found. Based on the owner’s statement, there have not been bad relations with the neighbours, and the garden is surrounded by a high wall, so the probability of intentional poisoning is very low.

### SAMPLE EXAMINATION

**Figure 1:**
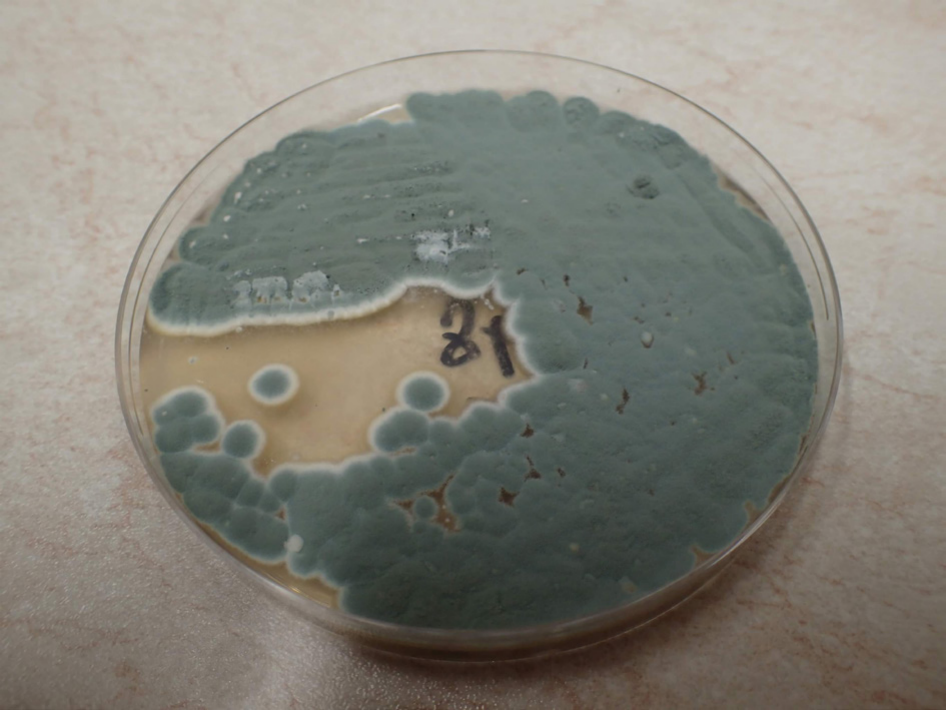
Sabouraud Agar with cultivated *Penicillium* sp. (photo by Vera Vaibarova)

**Figure 2:**
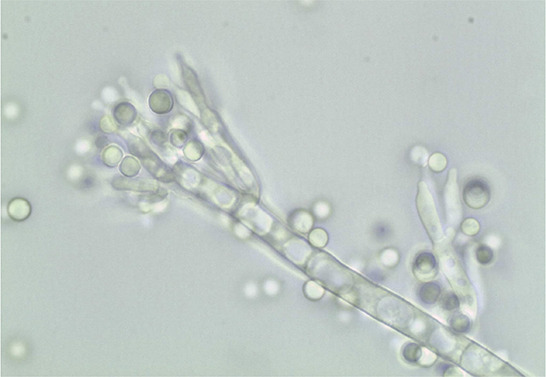
Conidiophore of *Penicillium* sp*.* on microscopic examination; original magnification, 400 × (photo by Vera Vaibarova)

Both the vomit and the pieces of the compost samples were sent for mycological examination to the Department of Infectious Diseases and Microbiology at the University of Veterinary Sciences of Brno (Czech Republic). Samples were cultivated on Sabouraud dextrose agar (Thermo Fisher Scientific, Basingstoke, Hampshire, UK) at 25 °C for 5 days. In the vomit sample, a pure culture of fast-growing fungus was isolated. Identification was performed by macroscopic and microscopic examination of typical structures. Isolate was identified as *Penicillium * sp*.* (Figure 1) and its conidiophores were detected on microscopic examination (Figure 2). Mycological cultivation of the compost sample was negative. The sample of the vomit and the growth medium with *Penicillium* sp*.* were subsequently sent for analysis of mycotoxins to the Metrological and Testing Laboratory of the University of Chemistry and Technology of Prague (Czech Republic). Multi-detection ultra-high performance liquid chromatography-tandem mass spectrometry (UHPLC-MS/MS) method targeting 56 toxic secondary metabolites of various fungi was utilised (Dzuman et al. 2014). Briefly, QuEChERS like extraction was used for analytes isolation, the acetonitrile extract was then separated on C18 chromatographic UHPLC column, mass spectrometer with orbitrap mass analyser was used as a detector. The standard addition method was employed for detected mycotoxins quantification. In both samples, significant concentrations of penitrem A, paxilline and roquefortine C were determined (Table 1, Table 2). In addition to these mycotoxins possessing tremorgenic properties, alternariol, alternariol-methylether, beauvericin, enniatin A and A1, enniatin B and B1, mycophenolic acid and cyclopiazonic acid were also present in the vomit sample (Table 1).

**Table 1:** Results of UHPLC-MS/MS analysis of the vomit sample

Analyte	Concentration (μg/kg)
Alternariol	190
Alternariol-methylether	5
Beauvericin	2 190
Cyclopiazonic acid	70
Enniatin A	1
Enniatin A1	17
Enniatin B	37
Enniatin B1	50
Mycophenolic acid	336
Paxilline	586
Penitrem A	41 900
Roquefortine C	70 200

UHPLC-MS/MS = ultra-high performance liquid chromatography tandem mass spectrometry

**Table 2:** Results of UHPLC-MS/MS analysis of the growth medium with *Penicillium* sp.

Analyte	Concentration (μg/kg)
Paxilline	241
Penitrem A	31 000
Roquefortine C	35 700

UHPLC-MS/MS = ultra-high performance liquid chromatography tandem mass spectrometry

### DIAGNOSIS

According to the history, clinical signs, and results of the laboratory analyses we can confirm the dog was poisoned by tremorgenic mycotoxins paxilline, penitrem A and roquefortine C. As *Penicillium* sp*.* was not cultivated in the sample of the compost, the exact source of the poisoning could not be proven. Due to the fact that the sample was taken one week later and the mould may have been irregularly distributed in the compost, we cannot exclude there had been a part of the compost containing *Penicillium * sp. present on the day when the dog was poisoned.

## DISCUSSION

In this study, a case of tremorgenic mycotoxin poisoning in a dog was reported with a high suspicion of a part of compost as a source of the mycotoxins. Other case reports describe intoxication caused by mouldy dog food, rotten apples, waste, cream cheese, and compost as well (Boysen et al. 2002; Walter 2002; Young et al. 2003; Eriksen et al. 2010). The diagnosis was confirmed by mycological and chromatographic analyses of the vomit sample where tremorgenic mycotoxins penitrem A and roquefortine C were detected at the highest amounts, but also another tremorgenic mycotoxin paxilline was present at a significant concentration. In the previous studies, penitrems and roquefortine were determined in mouldy food or gastric contents of poisoned dogs (Boysen et al. 2002; Walter 2002; Young et al. 2003; Eriksen et al. 2010). According to the literature, *Penicillium* species are most often incriminated in producing tremorgenic mycotoxins; the most common are penitrem A and roquefortine C (Schell 2000). As fungi produce multiple toxins simultaneously, a larger number of mycotoxins is usually involved in the intoxication, usually acting in synergy with one another (Gupta 2007; Tiwary et al. 2009; Svobodova et al. 2017; Evans and Gupta 2018). In our study, there were also several mycotoxins present such as alternariol, beauvericin, enniatins, mycophenolic acid, and cyclopiazonic acid in the respective sample. Although the acute toxicity of these mycotoxins seems to be low, they could contribute to the clinical symptoms due to their cytotoxicity (Schneweis et al. 2000; Fraeyman et al. 2017). Cyclopiazonic acid is known for its neurotoxicity including tremors and convulsions (Burdock and Flamm 2000). In the vomit sample, it was present in lower concentration compared to tremorgenic mycotoxins, nevertheless, it could strengthen their neurotoxic activity.

Clinical signs of intoxication by tremorgenic mycotoxins include diminished activity, muscle tremor, convulsions, ataxia, nystagmus, mydriasis, hypersalivation, tachycardia, hyperthermia and hyperaemia of mucosa, vomiting, and diarrhoea are often observed (Cole and Cox 1981; Valdes et al. 1985). Moreover, noise and excessive manipulation of the patient can lead to the worsening of its condition (Gupta 2007; Barker et al. 2013; Svobodova et al. 2017; Evans and Gupta 2018). In our study, miosis on both pupils was observed, which had also been described previously in tremorgenic mycotoxin poisoning cases (Nishiyama and Kuga 1986; Pino 2018). In the study done by Nishiyama and Kuga (1986), a potent stimulating effect of tremorgenic mycotoxin fumitremorgin A on the rabbit resulting in bilateral miosis was described. It seems that tremorgenic mycotoxins can have potent stimulating effects on the autonomic nervous system with possible involvement of parasympathetic hyperactivity. In most of the cases (Boysen et al. 2002; Walter 2002; Eriksen et al. 2010) there was hyperthermia present on admission apparently due to severe convulsions. Although generalised convulsions were present in our case, there were not severe enough to cause hyperthermia. First signs of intoxication appear approximately half an hour after ingestion of mouldy food. The intensity of symptoms depends on the dose of ingested mycotoxins and also on the ability of the patient to vomit out the ingested food containing mycotoxins. Similar clinical signs are also associated with intoxications by strychnine, metaldehyde, ethylene glycol, ivermectin, organophosphates, carbamates and xanthines. The differential diagnosis may include infectious diseases such as rabies or distemper (Barker et al. 2013). Because of the favourable epizootological situation in the country and the fact that the dog was regularly vaccinated and has not been recently injured by another animal, these two diseases were completely ruled out as possible causes of the symptoms. Furthermore, hypocalcaemia, hypoglycaemia, idiopathic tremor syndrome, hepatic encephalopathy, and cerebellar disorder may cause tremor as well (Barker et al. 2013). Because of the blood test results metabolic causes were excluded. In our case there was hypophosphataemia, hyperglycaemia, and thrombocytosis, the second analysis done a few hours later revealed neutrophilia and lymphocytopaenia which developed probably due to the acute stress. Hypophosphataemia probably resulted from vomiting. Slight variations in certain blood indicators may have been due to the fact that the analysis was performed on two different machines. The owner claimed they did not use any pesticides or paints in the household recently and the dog was denied approach to the house so the poisoning by heavy metals, methylxanthines, or food from a trash could be excluded. The owner had not administered any medication to the dog before. The dog did not have an approach to the garage and there was not any antifreeze present in the garden, so the poisoning by ethylene glycol was also excluded. Furthermore, ethylene glycol may cause hypocalcaemia, hypoglycaemia, hyperkalaemia, hyperphosphataemia and azotaemia (Rajagopal et al. 1977; Leth and Gregersen 2005; Schweighauser and Francey 2015) and any of these abnormalities were present in our case. We cannot completely rule out the possibility that somebody might have given the dog some of the listed substances during the owner’s absence, but the probability is very low based on the history and fencing of the object. The exact confirmation of tremorgenic mycotoxin poisoning lies in their detection in food residues, vomitus, stomach contents, urine, bile, and blood or in samples of the liver, kidneys, and cerebellum (Gupta 2007; Puschner 2009; Barker et al. 2013; Svobodova et al. 2017; Evans and Gupta 2018). Analytic methods include identification of the mould through the culture of the organism or detection of the toxins themselves, although the presence of mould does not necessarily mean mycotoxin is present at the same time. Liquid chromatography-mass spectrometry has been used to screen for specific toxins (Barker et al. 2013). In this case, vomit gained right before the onset of clinical signs was used for the analysis as a potentially substantial source of mycotoxins, and the mycological cultivation was used to see if there is mould present. To confirm the diagnosis, the sample was further analysed for tremorgenic mycotoxins including their quantification. Before any therapy is initiated, it is essential to prevent the animal from further ingestion of the suspected food. Since no specific medicament or antitoxin for tremorgenic mycotoxicosis is available, the treatment is symptomatic and it involves suppressing seizures, control of hyperthermia, and detoxification. In our case, activated charcoal was administered, with consequent administration of humic acids for decontamination. As there is evidence that the mycotoxins are excreted in bile, suggesting the possibility of hepatic recirculation (Barker et al. 2013), the detoxification was repeated. The fluids were administered throughout the whole hospitalization to maintain hydration and to correct electrolyte abnormalities. Because the dog continued vomiting during the night, maropitant, famotidine and later omeprazole were administered. For seizure suppression, diazepam was used. Benzodiazepines, e.g., diazepam, bind to the diazepin receptor and facilitates the GABAA receptor function, leading to decreased stimulation (Eriksen et al. 2010) and they are usually recommended initially to control seizures. According to the literature (Barker et al. 2013), there is some evidence that seizures caused by mycotoxins respond poorly to diazepam. If this is insufficient, methocarbamol or barbiturates should be administered (Young et al. 2003). Many studies describe rapid improvement after suppressing the seizures with barbiturates (Boysen et al. 2002; Walter 2002), but there is a risk of depression in the respiratory centre and sometimes ventilation of the patient is needed. In our case there was an improvement after the bolus of diazepam, nevertheless, repeated administration was required. Primary prevention is eliminating the risk of ingestion of the source of intoxication by the animal. Clinical signs usually disappear in a few days, some cases report the persistence of lower-intensity signs months, even years, after digestion. The toxic dose of tremorgenic mycotoxins for dogs and cats has not yet been determined. In high doses, these mycotoxins occasionally cause death, and late initiation of treatment may lead to the same outcome (Gupta 2007; Barker et al. 2013; Svobodova et al. 2017; Evans and Gupta 2018). In this study, the dog recovered rapidly without further complications, similar to the case reported by Boysen et al. (2002) probably because of early vomiting of the contaminated material and subsequent rapid repeated detoxification and intensive supportive therapy.

The aim of this study was to point out the possibility of using a comprehensive diagnosis of tremorgenic mycotoxin poisoning. Fungal cultivation was used as the primary screening method, the actual diagnosis was subsequently confirmed by a highly sensitive chromatographic method enabling the quantification of more than fifty fungal metabolites. In addition to penitrem A and roquefortine C, well-known tremorgenic mycotoxins, paxilline was also detected at significant concentrations. Since the development of clinical symptoms often occurs due to the action of several different mycotoxins, this chromatographic method appears to be very beneficial not only for diagnostics in veterinary practice.

## References

[R1] Arp LH, Richard JL. Experimental intoxication of guinea pigs with multiple doses of the mycotoxin, penitrem A. Mycopathologia. 1981 Jan;73(2):109-13. 7219513 10.1007/BF00562600

[R2] Barker AK, Stahl C, Ensley SM, Jeffery ND, Decus D. Tremorgenic mycotoxicosis in dogs. Compend Contin Educ Vet. 2013 Feb;35(2):E2. 23532902

[R3] Boysen SR, Rozanski EA, Chan DL, Grobe TL, Fallon MJ, Rush JE. Tremorgenic mycotoxicosis in four dogs from a single household. J Am Vet Med Assoc. 2002 Nov;221(10):1441-4. 12458614 10.2460/javma.2002.221.1441

[R4] Burdock GA, Flamm WG. Safety assessment of the mycotoxin cyclopiazonic acid. Int J Toxicol. 2000 May;19(3):195-218.

[R5] Cole RA, Cox RH. Handbook of toxic fungal metabolites. New York, USA: Academic Press; 1981.

[R6] Dzuman Z, Zachariasova M, Lacina O, Veprikova Z, Slavikova P, Hajslova J. A rugged high-throughput analytical approach for the determination and quantification of multiple mycotoxins in complex feed matrices. Talanta. 2014 Apr;121:263-72. 24607137 10.1016/j.talanta.2013.12.064

[R7] Eriksen GS, Jaderlund KH, Moldes-Anaya A, Schonheit J, Bernhoft A, Jaeger G, Rundberget T, Skaar I. Poisoning of dogs with tremorgenic Penicillium toxins. Med Mycol. 2010 Feb;48(1):188-96. 19886763 10.3109/13693780903225821

[R8] Evans TJ, Gupta RC. Veterinary toxicology. 3^rd^ ed. Amsterdam, The Netherlands: Academic Press; 2018. 1238 p.

[R9] Fraeyman S, Croubels S, Devreese M, Antonissen G. Emerging fusarium and alternaria mycotoxins: Occurrence, toxicity and toxicokinetics. Toxins. 2017 Jul;9(7):228. 28718805 10.3390/toxins9070228 PMC5535175

[R10] Gupta RC. Veterinary toxicology basic and clinical principles. Amsterdam, The Netherlands: Elsevier; 2007. 1201 p.

[R11] Leth PM, Gregersen M. Ethylene glycol poisoning. Forensic Sci Int. 2005 Dec 20;155(2-3):179-84. 16226155 10.1016/j.forsciint.2004.11.012

[R12] Nishiyama M, Kuga T. Pharmacological effects of the tremorgenic mycotoxin fumitremorgin A. Jpn J Pharmacol. 1986 Apr;40(4):481-9. 3735799 10.1254/jjp.40.481

[R13] Pino J. Heat stroke secondary to tremorgenic mycotoxicosis in an Australian Shepherd [dissertation]. Ithaca, New York, USA: Cornell University; 2018. 28 p.

[R14] Puschner B. Penitrem A and roquefortine. In: Plumlee KH, editor. Clinical veterinary toxicology. St. Louis, MO: Mosby; 2009. p. 258-9.

[R15] Rajagopal G, Venkatesan K, Ranganathan P, Ramakrishnan S. Calcium and phosphorus metabolism in ethylene glycol toxicity in rats. Toxicol Appl Pharmacol. 1977 Mar;39(3):543-7. 854928 10.1016/0041-008x(77)90145-4

[R16] Schell MM. Tremorgenic mycotoxin intoxication. Vet Med-Czech. 2000;95:285-6.

[R17] Schneweis I, Meyer K, Hormansdorfer S, Bauer J. Mycophenolic acid in silage. Appl Environ Microbiol. 2000 Aug;66(8):3639-41. 10919834 10.1128/aem.66.8.3639-3641.2000 PMC92198

[R18] Schweighauser A, Francey T. Ethylene glycol poisoning in three dogs: Importance of early diagnosis and role of hemodialysis as a treatment option. Schweiz Arch Tierheilkd. 2016 Feb;158(2):109-14. 27145686 10.17236/sat00051

[R19] Selala MI, Daelemans F, Schepens PJ. Fungal tremorgens: The mechanism of action of single nitrogen containing toxins – A hypothesis. Drug Chem Toxicol. 1989 Sep-Dec;12(3-4):237-57. 2698801 10.3109/01480548908999156

[R20] Sobotka TJ, Brodie RE, Spaid SL. Neurobehavioral studies of tremorgenic mycotoxins verruculogen and penitrem A. Pharmacology. 1978;16(5):287-94. 643898 10.1159/000136781

[R21] Svobodova Z, Modra H, Herzig I, Skaloud J. Veterinarni toxikologie v klinicke praxi [Veterinary toxicology in clinical practice]. 2^nd^ ed. Prague, Czech Republic: Profi Press; 2017. 280 p. Czech.

[R22] Tiwary AK, Puschner B, Poppenga RH. Using roquefortine C as a biomarker for penitrem A intoxication. J Vet Diagn Invest. 2009 Mar;21(2):237-9. 19286504 10.1177/104063870902100210

[R23] Valdes JJ, Cameron JE, Cole RJ. Aflatrem: A tremorgenic mycotoxin with acute neurotoxic effects. Environ Health Perspect. 1985 Oct;62:459-63. 2867895 10.1289/ehp.8562459 PMC1568710

[R24] Walter SL. Acute penitrem A and roquefortine poisoning in a dog. Can Vet J. 2002 May;43(5):372-4. 12001505 PMC339273

[R25] Young KL, Villar D, Carson TL, Ierman PM, Moore RA, Bottoff MR. Tremorgenic mycotoxin intoxication with penitrem A and roquefortine in two dogs. J Am Vet Med Assoc. 2003 Jan 1;222(1):52-3. 12523480 10.2460/javma.2003.222.52

